# Microbiological analysis of endotracheal aspirate and endotracheal tube cultures in mechanically ventilated patients

**DOI:** 10.1186/s12890-019-0926-3

**Published:** 2019-08-27

**Authors:** Lijuan Shen, Fei Wang, Junfeng Shi, Weixin Xu, Tingting Jiang, Huifang Tang, Xiuwen Yu, Hao Yin, Shanyou Hu, Xiao Wu, Siu Kit Chan, Jie Sun, Qing Chang

**Affiliations:** 1 Department of Clinical Laboratory, Jiading District Central Hospital Affiliated Shanghai University of Medicine & Health Sciences, No.1, Chengbei Rd, Jiading District, Shanghai, 201800 China; 2 Department of Critical Care Medicine, Jiading District Central Hospital Affiliated Shanghai University of Medicine & Health Sciences, Shanghai, 201800 China; 3 Shanghai Key Laboratory for Molecular Imaging, Shanghai University of Medicine & Health Sciences, Shanghai, 301318 China; 4 Department of Pharmacology, Zhejiang University, School of Basic Medical Sciences, Hangzhou, 310058 China; 5 Emergency Department, Jiading District Central Hospital Affiliated Shanghai University of Medicine & Health Sciences, Shanghai, 201800 China; 6 Clinical Research Center, Jiading District Central Hospital Affiliated Shanghai University of Medicine & Health Sciences, No.1, Chengbei Rd, Jiading District, Shanghai, 201800 China

**Keywords:** Consistency analysis, Endotracheal tube, Eracheal aspirate, Mechanical ventilation, Microorganism, Clinical value

## Abstract

**Background:**

To compare the microbiological culture within endotracheal aspirate specimens (ETAs) and endotracheal tube specimens (ETTs) in patients undergoing mechanical ventilation (MV) by statistical tools.

**Method:**

ETAs and ETTs from a total number of 81 patients, who were undergoing MV at the intensive care unit (ICU) of Jiading Central Hospital Affiliated Shanghai University of Medicine & Health Sciences from September 1st, 2017 to August 31st, 2018, were collected for microbiological culture analysis. Correlation of ETAs and ETTs cultures were obtained by Spear-men correlation analysis, while the consistency of the two specimens was determined by Kappa analysis and principal component analysis (PCA).

**Results:**

Microbiological culture from both ETAs and ETTs showed that *Acinetobacter baumannii*, *Pseudomonas aeruginosa*, *Staphylococcus aureus*, and *Klebsiella pneumoniae* were the main pathogens, with Spear-man correlation coefficients of 0.676, 0.951, 0.730 and 0.687 respectively (all *P* < 0.01), and the overall Spear-man correlation coefficient is 0.757 (P < 0.01). This result shows that two samples were positively correlated. Kappa analysis also revealed high consistency of the microbial culture results from the ETAs and the ETTs (overall κ = 0.751, P < 0.01). The κ values for the four bacteria detected were 0.670, 0.949, 0.723, and 0.687, respectively (all *P* < 0.001). PCA also revealed high similarity.

**Conclusion:**

Combining microbiological culture and statistical analysis of samples collected from 81 patients who were undergoing MV in ICU, we showed that microbe found in the ETAs had high similarity with that found in the ETTs which collected at the end of the catheters. In clinical practice, ETAs analysis is easily accessible meanwhile provides a valuable information for MV patients.

**Electronic supplementary material:**

The online version of this article (10.1186/s12890-019-0926-3) contains supplementary material, which is available to authorized users.

## Background

Lower respiratory tract infections in mechanically ventilated patients are a primary cause of mortality remains stubbornly high in intensive-care units (ICU) [1–4]. A previous study has shown that well-timed and accurate identification of the microorganisms residing in the lungs could effectively diminish the mortality of such cases [5]. Because of this, microbiological culture analysis on patients’ endotracheal aspirate specimens (ETAs) is commonly used in clinical practices for determining the presence and the stain of the residing pathogen. However, it has been suggested that the microbiological culture analysis on ETAs alone could not distinguish the colonization from the infection [6, 7].

It was shown that there is a very high probability for biofilm to grow at the end of an endotracheal tubes intubated in patients receiving mechanical ventilation (MV) [8], in which corresponds to the change of the micro-environment inside the lower respiratory tract and enhance infection [9–11]. It was, therefore, suggested that the endotracheal tube specimens (ETTs) is more appropriate for etiological examination than ETAs [12]. Meanwhile, collection of ETTs from MV patients is neither clinically practical nor of sufficient simplicity [13]. This leads to the discussion whether ETTs or ETAs is more suitable for the etiology of infection of MV patients.

Recently, Pan et al. compared the pathogenic microbiology between ETAs and ETTs of patients on MV in pediatric ICU [14]. Their study showed that both results of microbiological culture are highly similar. It is interesting to ask if we could extend this conclusion to adult patients on MV. In this clinical study, we collected ETAs and ETTs from 81 adult patients on MV in ICU and performed microbiological culture analysis. By Spear-man correlation analysis, Kappa analysis as well as principle component analysis (PCA), we showed that microbial data in ETAs and ETTs are highly conserved. Therefore, we believe that it is not necessary to use ETTs instead of ETAs of clinical practices.

## Methods

### Subjects

Inclusion criteria: Patients treated with MV at the ICU of Jiading District Central Hospital Affiliated Shanghai University of Medicine & Health Sciences. All intubations were preformed according to “American Journal of respiratory and critical care medicine” [15]. Exclusion criteria: (1) Refusal by the patients’ guardian; (2) Patients showing inconsistency in the results of Gram staining and microbiological culture; (3) Other factors (Patients with no ETAs, ETTs contamination during extubation or transportation, etc.).

Diagnostic criteria for lower respiratory tract infections: (1) Patients with body temperature more than 38 °C, (2) leukocytosis, (3) microbiological culture showed a positive result, (4) lung density in physical examination in association with the presence or changes in radiographic infiltration [16, 17].

### Information and sample collection

General information (gender, age) and past medical history (hypertension, diabetes, etc.) of patients were collected, and the Acute Physiology and Chronic Health Evaluation II (APACHE-II) score was calculated on the day of admission. The length of hospital stay of each patient were tracked until leaving the ICU or death.

ETTs were obtained immediately after extubation. Roughly 1 cm of the distal end of the ETTs was cut for microbiological culture analysis. ETAs was extracted by a steriled suction tube, which inserted into the lower respiratory tract through a catheter.

### Materials and instruments

Blood agar plates, MacConkey plates, and chocolate agar plates were provided by Shanghai Yihua Biotechnology Co., Ltd. (Shanghai, China). The VITEK 2 compact automatic bacterial identification and drug susceptibility analysis systems (bioMérieux, Marcy-l’Étoile, France) was used. *Staphylococcus aureus* (ATCC29213) and *Pseudomonas aeruginosa* (ATCC27853) were purchased from the Shanghai Clinical Laboratory Center as the quality control strains.

### Microbiological culture analysis

Microbiological culture analysis of ETAs and ETTs collected was preformed according to the CSLI method [18]. Briefly, part of the ETAs and ETTs collected was subjected to Gram staining, and the rest of the samples were immediately incubated onto a blood agar plate, a MacConkey plate, and a chocolate agar plate. All plates were incubated at 35 °C with 5% CO_2_ for 18–24 h before interpretation. Pure cultures and dominant pathogens were identified by the VITEK2 systems. The result of microbiological culture was included only when the dominant strains were consistent with the Gram staining result.

### Statistical analysis

All statistical analyses were performed in the R environment [19]. Data were summarized as mean ± standard deviation (SD) or percentage. For the data of duration of ventilation days, the median was used, indicated as M (Q1~Q3). One-way analysis of variance and chi-square test were used for statistical analysis. Spear-man correlation analysis, coincidence rate, kappa coefficient and PCA were performed to analyze the results of microbiological culture from the two kinds of samples.

## Results

### Study population

**Fig. 1:**
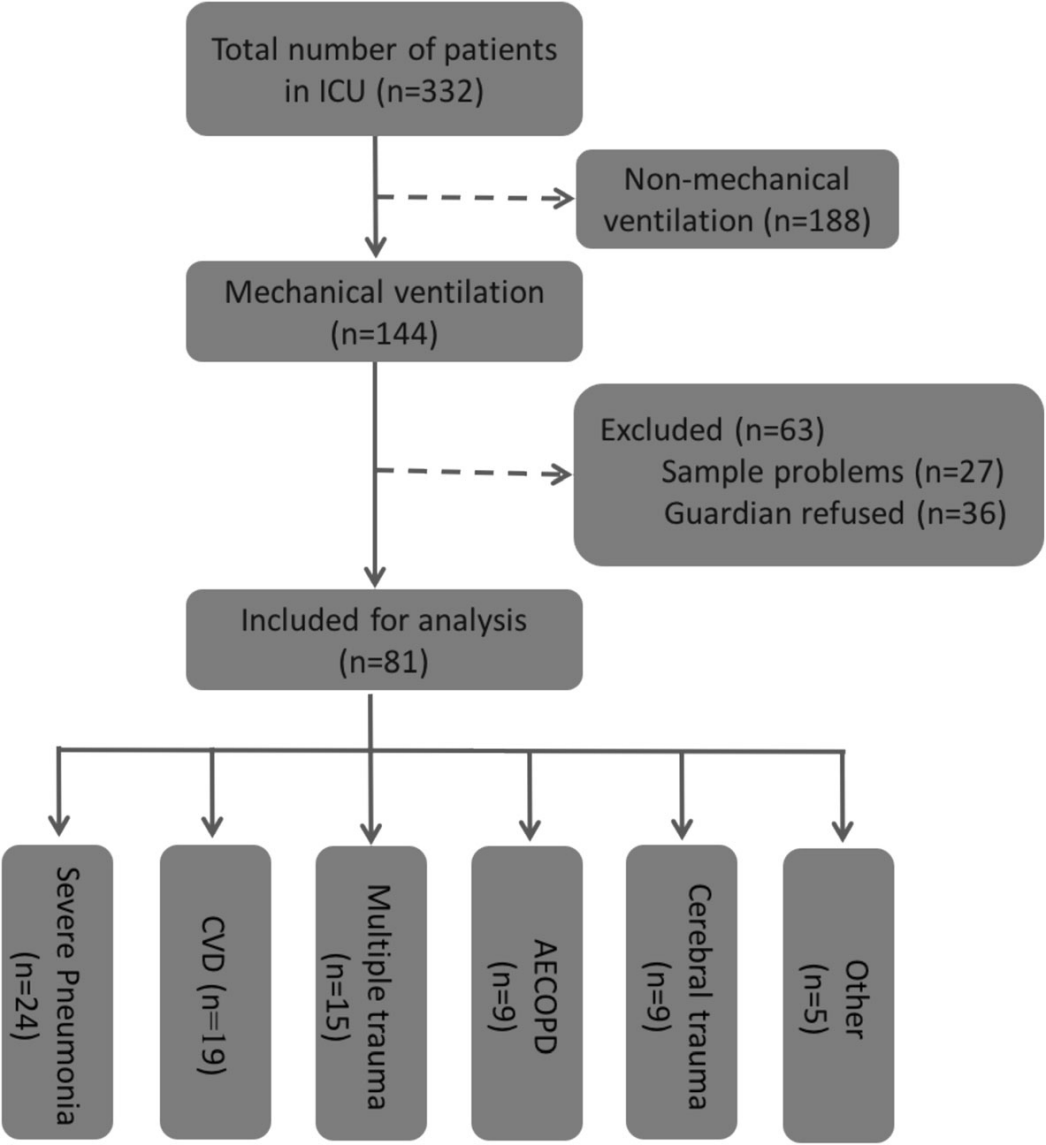
Enrolled case Flowchart. CVD:cerebrovascular disease; AECOPD:Acute exacerbation of chronic obstructive pulmonary disease

There were 332 patients admitted to the ICU of Jiading District Central Hospital Affiliated Shanghai University of Medicine & Health Sciences from September 1st, 2017 to August 31st, 2018, of which 144 were treated with MV. 81 of them matched the criteria and were included in this study. The reasons of hospitalization were summarized as followed: severe pneumonia (*n* = 24), severe cerebrovascular disease (*n* = 19), multiple injuries (*n* = 15), acute exacerbation of chronic obstructive pulmonary disease (*n* = 9), cerebral trauma (n = 9), and others (*n* = 5; Fig. 1). The mean age of patients was 62.1 ± 19.7 years, in which 63 patients were males (77.8%) and 18 patients were females (22.2%; Table 1). ETTs was extubated when: (i) Patients recovered (*n* = 49, 60.5%); (ii) change of ETTs (*n* = 3, 3.7%); and (iii) patients succumbed to mortality (*n* = 29, 35.8%).

**Table 1:** Characteristics of patients

Patient characteristics (*n* = 81)	
Male/female	63/18
Age (years): mean ± SD; (range)	62.11 ± 19.70 (20.0–96.0)
Hypertension [n(%)]	33 (40.7)
Diabetes mellitus [n(%)]	13 (16.0)
APACHEII (score): mean ± SD; (range)	25.10 ± 5.98; (10–43)
LRTI [n(%)]	41 (50.6)
Duration of ventilation days: M(Q1–Q3); (range)	5 (3–8); (0.5–87.0)

APACHE (Acute Physiology and Chronic Health Evaluation) II; LRTI: Lower respiratory tract infection

### Microbiological culture results

**Fig. 2:**
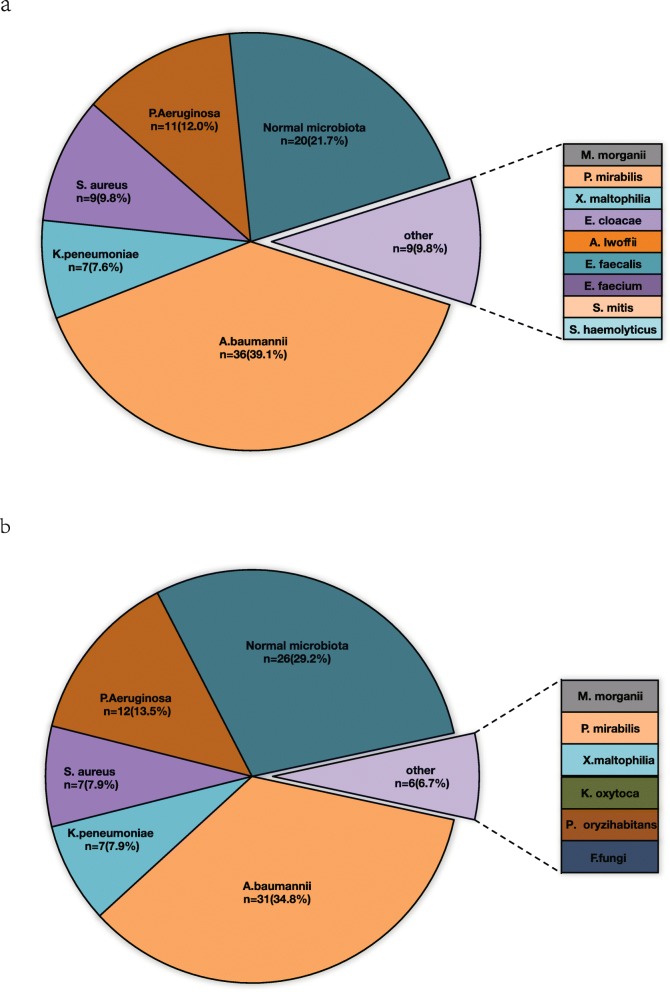
Microbial distribution pie chart. ETTs distribution pie chart (2a); ETAs distribution pie chart (2b). *A.baumannii:Acinetobacter baumannii; K.peneumoniae:Klebsiella pneumoniae; P.Aeruginosa: Pseudomonas aeruginosa; S. aureus: Staphylococcus aureus; E. cloacae: Enterobacter cloacae; S. mitis:Streptococcus mitis; A.lwoffii:Acinetobacter lwoffii; E. faecalis: Enterococcus faecalis; E.faecium: Enterococcus faecium; P. mirabilis:Proteus mirabilis; S. haemolyticus:Staphylococcus haemolyticus; S. maltophilia:Stenotrophomonas maltophilia ; M. morganii: Morganella morganii; K. oxytoca: Klebsiella oxytoca; P. oryzihabitans: Pseudomonas oryzihabitans; X. maltophilia: Xanthomonas maltophilia; F. fungi: Filamentous fungi*

Within 81 ETTs, 61 of them (75.3%) grew one (50) or two (11) major populations of pathogenic bacteria, while the other 20 samples grew single major population of normal respiratory track flora. i.e. A total number of 92 major populations of cells were observed, in which 72 of them were pathogenic bacteria and 20 were normal respiratory track flora. Bacterial identification results showed that the main pathogens observed were *A. baumannii* (39.1%, 36/92), *P. aeruginosa* (12.0%, 11/92), *S. aureus* (9.8%, 9/92), and *K. pneumoniae* (7.6%, 7/92) respectively (Fig. 2a).

For ETAs analysis, 57 specimens (70.4%) grew pathogenic bacteria as major cell population, while 24 of them grew normal respiratory track flora as major population of cell. Totally 89 populations of cells were observed, of which 63 were pathogenic bacteria and 26 were normal respiratory track flora. The main pathogenic bacteria stains were *A. baumannii* (34.8%, 31/89), *P. aeruginosa* (13.5%, 12/89), *S. aureus* (7.9%, 7/89), and *K. pneumoniae* (7.9%, 7/89; Fig. 2b).

### Correlation analysis

Spear-man correlation analysis showed that in general the results of microbiological culture from ETAs were positively correlated with those from ETTs (Spear-man γ = 0.757, *P* < 0.01). In addition, *P. aeruginosa*, normal respiratory flora, *S. aureus*, *K. pneumoniae*, and *A. baumannii* showed positively correlation between two kinds of specimens (Spear-man γ = 0.951, 0.757, 0.730, 0.687, 0.676, all P < 0.01). Lower respiratory tract infection rate was positively correlated with age (Spear-man γ = 0.561, P < 0.01). The presence of normal respiratory flora is negatively correlated with the growth of pathogens in either ETTs (Spear-man γ = − 1, P < 0.01) or ETAs (Spear-man γ = − 0.970, P < 0.01) samples (Table 2) as expected. Meanwhile, we analyzed factors including diabetes mellitus, hypertension, duration of ventilation days, APACHE-II and the cause of hospital admission with the results of microbiological culture from the two samples types (Additional file 1: Table S1, S2), the results showed low correlation between those factors and the type and diversity of bacterial species found within either type of the two samples.

**Table 2:** Correlation analysis of two specimens and variables

	Sex	Age	LRTI	T-Ab	A-Ab	T-Kpn	A-Kpn	T-SA	A-SA	T-PA	A-PA	T-NM	A-NM	T-other	A-other	T-SUM	A-SUM
Sex	1																
Age	–	1															
LRTI	–	0.563^**^	1														
T-Ab	–	–	–	1													
A-Ab	–	–	–	0.676^**^	1												
T-Kpn	–	–	–	–	–	1											
A-Kpn	–	–	–	–	–	0.687^**^	1										
T-SA	–	–	–	–	–	–	–	1									
A-SA	–	–	–	–	–	–	–	0.73^**^	1								
T-PA	–	–	–	–	–	–	–	–	–	1							
A-PA	–	–	–	–	–	–	–	–	–	0.951^**^	1						
T-NM	–	–	–	−0.512^**^	–	–	–	–	–	–	–	1					
A-NM	–	–	–	–	−0.496^**^	–	–	–	–	–	–	0.782^**^	1				
T-other	–	–	–	–	–	–	–	–	–	–	–	–	–	1			
A-other	–	–	–	–	–	–	–	–	–	–	–	–	–	0.45^**^	1		
T-SUM	–	–	–	0.512^**^	–	–	–	–	–	–	–	−1^**^	−0.782^**^	–	–	1	
A-SUM	–	–	–	0.417^**^	0.511^**^	–	–	–	–	–	–	−0.757^**^	−0.97^**^	–	–	0.757^**^	1

**: *P* < 0.01

*LRTI* Lower respiratory tract infection, *Ab Acinetobacter baumannii*, *Kpn Klebsiella pneumoniae*, *PA Pseudomonas aeruginosa*, *SA Staphylococcus aureus*, *NM* Normal microbiotas, *T* Endotracheal tube specimens, *A* endotracheal aspirate specimens

### Comparison of microbial data between both specimens

The comparison between culture data of ETAs and ETTs showed that the total microbial coincidence rate was 96.7%. The coincidence rate of *K. pneumoniae*, *P. aeruginosa*, and *A. baumannii* were 100, 90.9, and 86.1%, respectively (Table 3).

**Table 3:** Convergence rate of number of strains detected in two specimens

	ETTs	ETAs	Coincidence rate (%)
Total	92	87	96.7
Gram-negative	58	54	72.9
* A. baumannii*	36	31	86.1
* P. aeruginosa*	10	11	90.9
* K. pneumoniae*	7	7	100
Other	4	6	66.7
Gram-positive	14	7	50.0
* S. aureus*	9	7	77.8
Other	5	0	0
Normal microbiota	20	26	76.9

*ETTs* Endotracheal tube specimens, *ETAs* Endotracheal aspirate specimens, *A. baumannii Acinetobacter baumannii*, *K. pneumoniae Klebsiella pneumoniae*, *P. aeruginosa Pseudomonas aeruginosa*, *S. aureus Staphylococcus aureus*

Kappa analysis showed that microbial data of ETAs and ETTs were highly conserved in general (κ = 0.751); The κ values of *P. aeruginosa*, *S. aureus*, *K. pneumoniae*, and *A. baumannii* were 0.949, 0.723, 0.687, and 0.670, respectively (all *P* < 0.001).

**Fig. 3:**
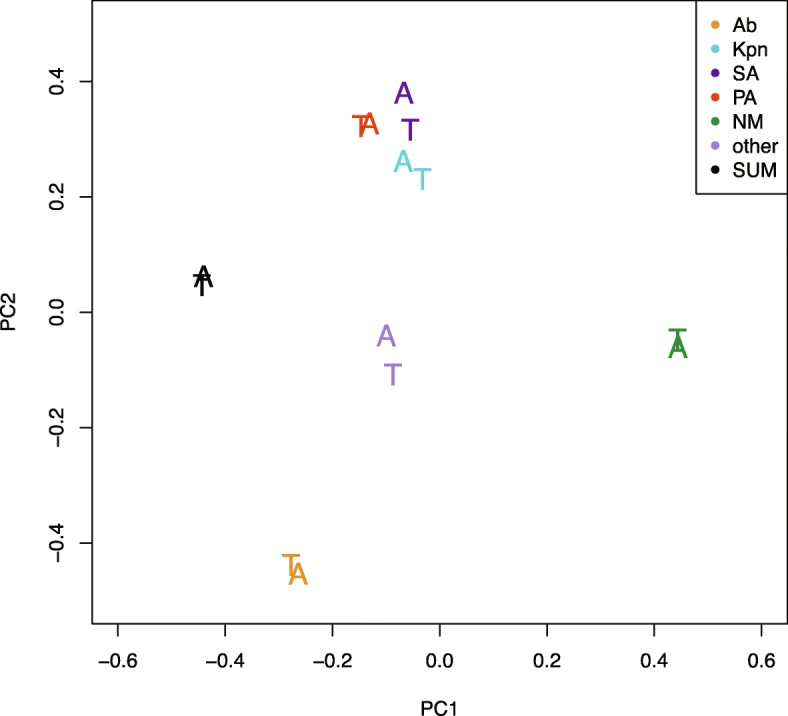
PCA was performed to evaluate the results of microbial culture from ETAs and ETTs. Ab:*Acinetobacter baumannii*; Kpn:*Klebsiella pneumoniae*; PA:*Pseudomonas aeruginosa*; SA:*Staphylococcus aureus*; NM:Normal microbiotas; T:Endotrcheal tube specimens ;A:Endotracheal aspirate specimens

PCA was performed and the result was as shown in Fig. 3. Each pathogen and the overall culture results showed a high degree of similarity.

## Discussion

In this study, we compared the microbiological data between ETTs and ETAs. Statistical tools (Spear-man correlation analysis, Kappa analysis and PCA) were applied and the results showed that the microbial components of ETAs and ETTs were highly conserved.

Microbial data showed that Gram negative pathogenic bacteria *A. baumannii, K. pneumonia* and *P. aeruginosa* were observed most frequently, which conserved with several recent studies on ETAs microbial components of ETAs [5, 7]. A detailed study suggested that the multi-resistant property of these Gram negative pathogens is a key for the high prevalence of biofilm of ETTs [13, 20]. These pathogens are often related to the infection of MV patients in ICU [8].

By directly counting the match rate, Gil-Perotin et al. showed that there was only 56% of similarity between ETAs and ETTs [10]. And by excluding *Candida* spp., which was considered to prefer attaching to the mucosa rather than the prosthesis, the match rate was 69%. The *Candida* spp. excluded match rate indicated that the microbial components of ETTs and ETAs should be similar, which is conserved to our conclusion.

Both Spear-man correlation coefficient (Spear-man γ = 0.757) and Kappa analysis showed that the ETAs is highly correlated to the ETTs in general, with κ = 0.751. The partially inconsistency is possibly due to: (1) The uneven distribution of microbial colonies due to the limited movement and cough reflex of patients undergoing MV [12, 21], (2) Microbials have higher resistance to antibiotics when forming biofilm on ETTs than suspending in ETAs, causing the differences in the microbial component between ETAs and ETTs [10, 22].

In this study, we demonstrated that the microbial components of ETAs and ETTs are highly correlated through microbiological culture analysis and statistical tools. The sample collection processes from MV patients could be formidable in clinical practices [13, 23]. And yet, a timely determination of the pathogen is crucial for proper treatment and therefore eventually reduce mortality [8]; and this become even more important when the function of immune system of patients decreases with ages [1], resulting an increase of infection rate. Due to the reasons mentioned above ETAs is more preferable for its accessibility in clinical practice, while its accuracy is highly conserved with the ETTs. Moreover, Hashemi et al. suggested that Gram stains of the ETAs help to establish the presence of inflammation based on numbers of polymorphnuclear cells [24], and quantitative culture (bacterial growth ≥10^5^ CFU/mL) may provide a better standard for comparison and greater diagnostic specificity to discriminate between colonization and infection in lower airway [17].

To further confirm the consistency among microbial components of ETTs and ETAs, a larger sample size could be used, e.g. combining data from ICUs of different hospitals. Up to date microbiological culture analysis is still a major technique for inspection in the hospitals of China, introducing biomarkers and molecular diagnostic techniques could enhance the inspection processes.

## Conclusions

In summary, the microbial data of ETAs and ETTs of MV patients in the ICU are highly correlated. The ETAs is more preferable for etiological examination of lower respiratory tract infections in MV patients.

## Additional file

Additional file 1: **Table S1.** Correlation analysis of two specimens and variables. **Table S2.** Correlation analysis of between the cause of hospitalization and the type of bacterial species. (DOCX 40 kb)

## Data Availability

The datasets used and/or analyzed during the current study are available from the corresponding author on reasonable request.

## Abbreviations

*A. baumannii*/ Ab: *Acinetobacter baumannii*
*A. lwoffii*: *Acinetobacter lwoffii*
AECOPD: Acute exacerbation of chronic obstructive pulmonary disease
APACHE II: Acute Physiology and Chronic Health Evaluation II
CVD: Cerebrovascular disease
*E. cloacae*: *Enterobacter cloacae*
*E. faecalis*: *Enterococcus faecalis*
*E. faecium*: *Enterococcus faecium*
ETAs/ A: Endotracheal aspirate specimens
ETTs/ T: Endotracheal tube specimens
*F. fungi*: *Filamentous fungi*
ICU: Intensive care unit
*K. oxytoca*: *Klebsiella oxytoca*
*K. pneumoniae*/ Kpn: *Klebsiella pneumoniae*
LRTI: Lower respiratory tract infection
*M. morganii*: *Morganella morganii*
MV: Mechanical ventilation
NM: Normal microbiota
*P. aeruginosa*/ PA: *Pseudomonas aeruginosa*
*P. mirabilis*: *Proteus mirabilis*
*P. oryzihabitans*: *Pseudomonas oryzihabitans*
PCA: principal component analysis
*S. aureus*/ SA: *Staphylococcus aureus*
*S. haemolyticus*: *Staphylococcus haemolyticus*
*S. maltophilia*: *Stenotrophomonas maltophilia*
*S. mitis*: *Streptococcus mitis*
*X. maltophilia*: *Xanthomonas maltophilia*
