# Supplementary material for: Microbiological analysis of endotracheal aspirate and endotracheal tube cultures in mechanically ventilated patients
Source: BMC Pulm Med. 2019 Aug 27;19:162. doi: 10.1186/s12890-019-0926-3 (PMC6712863; doi:10.1186/s12890-019-0926-3)
Supplement: Supplementary file 1 — Table S1. Correlation analysis of two specimens and variables. Table S2. Correlation analysis of between the cause of hospitalization and the type of bacterial species. (DOCX 40 kb) [file 12890_2019_926_MOESM1_ESM.docx]

**Table S1.** Correlation analysis of relevant variables and specimens

|  | Sex | Age | Prog | LRTI | HBP | DM | DVD | AP | T-Ab | A-Ab | T-Kpn | A-Kpn | T-SA | A-SA | T-PA | A-PA | T-NM | A-NM | T-other | A-other | T-SUM | A-SUM |
| --- | --- | --- | --- | --- | --- | --- | --- | --- | --- | --- | --- | --- | --- | --- | --- | --- | --- | --- | --- | --- | --- | --- |
| Sex | 1 |  |  |  |  |  |  |  |  |  |  |  |  |  |  |  |  |  |  |  |  |  |
| Age | - | 1 |  |  |  |  |  |  |  |  |  |  |  |  |  |  |  |  |  |  |  |  |
| Prog | - | - | 1 |  |  |  |  |  |  |  |  |  |  |  |  |  |  |  |  |  |  |  |
| LRTI | - | 0.563^**^ | - | 1 |  |  |  |  |  |  |  |  |  |  |  |  |  |  |  |  |  |  |
| HBP | - | - | - | - | 1 |  |  |  |  |  |  |  |  |  |  |  |  |  |  |  |  |  |
| DM | - | - | - | - | - | 1 |  |  |  |  |  |  |  |  |  |  |  |  |  |  |  |  |
| DVD | - | - | - | - | - | - | 1 |  |  |  |  |  |  |  |  |  |  |  |  |  |  |  |
| AP | - | - | - | - | - | - | - | 1 |  |  |  |  |  |  |  |  |  |  |  |  |  |  |
| T-Ab | - | - | - | - | - | - | - | - | 1 |  |  |  |  |  |  |  |  |  |  |  |  |  |
| A-Ab | - | - | - | - | - | - | - | - | 0.676^**^ | 1 |  |  |  |  |  |  |  |  |  |  |  |  |
| T-Kpn | - | - | - | - | - | - | - | - | - | - | 1 |  |  |  |  |  |  |  |  |  |  |  |
| A-Kpn | - | - | - | - | - | - | - | - | - | - | 0.687^**^ | 1 |  |  |  |  |  |  |  |  |  |  |
| T-SA | - | - | - | - | - | - | - | - | - | - | - | - | 1 |  |  |  |  |  |  |  |  |  |
| A-SA | - | - | - | - | - | - | - | - | - | - | - | - | 0.73^**^ | 1 |  |  |  |  |  |  |  |  |
| T-PA | - | - | - | - | - | - | - | - | - | - | - | - | - | - | 1 |  |  |  |  |  |  |  |
| A-PA | - | - | - | - | - | - | - | - | - | - | - | - | - | - | 0.951^**^ | 1 |  |  |  |  |  |  |
| T-NM | - | - | - | - | - | - | - | - | -0.512^**^ | - | - | - | - | - | - | - | 1 |  |  |  |  |  |
| A-NM | - | - | - | - | - | - | - | - | - | -0.496^**^ | - | - | - | - | - | - | 0.782^**^ | 1 |  |  |  |  |
| T-other | - | - | - | - | - | - | - | - | - | - | - | - | - | - | - | - | - | - | 1 |  |  |  |
| A-other | - | - | - | - | - | - | - | - | - | - | - | - | - | - | - | - | - | - | 0.45^**^ | 1 |  |  |
| T-SUM | - | - | - | - | - | - | - | - | 0.512^**^ | - | - | - | - | - | - | - | -1^**^ | -0.782^**^ | - | - | 1 |  |
| A-SUM | - | - | - | - | - | - | - | - | 0.417^**^ | 0.511^**^ | - | - | - | - | - | - | -0.757^**^ | -0.97^**^ | - | - | 0.757^**^ | 1 |

**: P < 0.01

HBP: Hypertension; DM: Diabetes mellitus; AP: Acute Physiology and Chronic Health Evaluation II; LRTI: Lower respiratory tract infection; DVD: duration of ventilation days; prog: prognosis; Ab: *Acinetobacter baumannii*; Kpn: *Klebsiella pneumoniae*; PA: *Pseudomonas aeruginosa*; SA: *Staphylococcus aureus*; NM: Normal microbiotas; T: Endotracheal Tube; A: Endotracheal aspirates

**Table S2.** Correlation analysis between the cause of hospitalization and the type of bacterial species

|  | SP | OC | CVD | CT | COPD | MT | T-Ab | A-Ab | T-Kpn | A-Kpn | T-SA | A-SA | T-PA | A-PA | T-NM | A-NM | T-other | A-other |
| --- | --- | --- | --- | --- | --- | --- | --- | --- | --- | --- | --- | --- | --- | --- | --- | --- | --- | --- |
| SP | 1 |  |  |  |  |  |  |  |  |  |  |  |  |  |  |  |  |  |
| OC | -0.166 | 1 |  |  |  |  |  |  |  |  |  |  |  |  |  |  |  |  |
| CVD | -0.359 | -0.142^*^ | 1 |  |  |  |  |  |  |  |  |  |  |  |  |  |  |  |
| CT | -0.229 | -0.091 | -0.196 | 1 |  |  |  |  |  |  |  |  |  |  |  |  |  |  |
| AECOPD | -0.229 | -0.091 | -0.196 | -0.125 | 1 |  |  |  |  |  |  |  |  |  |  |  |  |  |
| MT | -0.309 | -0.122 | -0.264 | -0.169 | -0.169 | 1 |  |  |  |  |  |  |  |  |  |  |  |  |
| T-Ab | 0.181 | -0.023 | --0.143 | -0.158 | 0.158 | -0.043 | 1 |  |  |  |  |  |  |  |  |  |  |  |
| A-Ab | 0.101 | -0.096 | -0.136 | -0.198 | 0.126 | 0.148 | 0.676^**^ | 1 |  |  |  |  |  |  |  |  |  |  |
| T-Kpn | -0.007 | 0.104 | 0.141 | 0.031 | -0.109 | -0.147 | -0.275 | -0.152 | 1 |  |  |  |  |  |  |  |  |  |
| A-Kpn | -0.007 | 0.104 | 0.037 | 0.171 | -0.109 | -0.147 | -0.098 | -0.242 | 0.687^**^ | 1 |  |  |  |  |  |  |  |  |
| T-SA | -0.057 | 0.073 | 0.082 | 0.125 | -0.125 | -0.067 | -0.237 | -0.198 | -0.109 | -0.109 | 1 |  |  |  |  |  |  |  |
| A-SA | -0.007 | 0.104 | 0.141 | 0.031 | -0.109 | -0.147 | -0.187 | -0.242 | -0.095 | -0.095 | 0.73^**^ | 1 |  |  |  |  |  |  |
| T-PA | 0.058 | 0.048 | -0.049 | -0.025 | -0.140 | 0.089 | -0.064 | -0.090 | -0.122 | 0.006 | -0.140 | 0.006 | 1 |  |  |  |  |  |
| A-PA | 0.034 | 0.037 | -0.067 | -0.037 | -0.147 | 0.159 | -0.093 | -0.114 | -0.128 | -0.005 | -0.147 | -0.005 | 0.951^**^ | 1 |  |  |  |  |
| T-NM | -0.183 | -0.147 | 0.156 | 0.071 | -0.020 | 0.096 | -0.512^**^ | -0.392 | -0.176 | -0.176 | -0.202 | -0.176 | -0.227 | -0.158 | 1 |  |  |  |
| A-NM | -0.169 | -0.162 | 0.168 | 0.126 | 0.039 | -0.018 | -0.398 | -0.496^**^ | -0.096 | -0.194 | 0.136- | -0.194 | -0.250 | -0.263 | 0.782^**^ | 1 |  |  |
| T-other | 0.201 | -0.091 | -0.196- | -0.125 | 0.125 | 0.034 | 0 | 0.045 | -0.109 | -0.109 | 0 | -0.109 | -0.025 | -0.037 | -0.202 | -0.136 | 1 |  |
| A-other | -0.185 | 0.104 | -0.170 | -0.109 | 0.031 | -0.034 | -0.079 | -0.152 | -0.095 | -0.095 | 0.031 | -0.095 | 0.006 | -0.005 | -0.176 | -0.194 | 0.45^**^ | 1 |

SP：severe pneumonia, OC: other cause, CVD: cerebrovascular disease, CT: cerebral trauma, COPD: acute exacervation of chronic obstructive pulmonary disease , MT: Mutiple trauma, Ab: *Acinetobacter baumannii*; Kpn: *Klebsiella pneumoniae*; PA: *Pseudomonas aeruginosa*; SA: *Staphylococcus aureus*; NM: Normal microbiotas; T: Endotracheal Tube; A: Endotracheal aspirates.
